# The relation between trust and the willingness of enrollees to receive healthcare advice from their health insurer

**DOI:** 10.1186/s12913-022-09016-9

**Published:** 2023-01-18

**Authors:** Frank J. P. van der Hulst, Anne E. M. Brabers, Judith D. de Jong

**Affiliations:** 1 Nivel, the Netherlands Institute for Health Services Research, Otterstraat 118-124, 3513 CR Utrecht, PO Box 1568, 3500 BN Utrecht, the Netherlands; 2 Maastricht University, PO Box 616, 6200 MD Maastricht, the Netherlands

**Keywords:** Health insurance, Trust, Healthcare advice, Healthcare system, Managed competition

## Abstract

**Background:**

In a healthcare system based on managed competition, it is important that health insurers are able to channel enrollees to preferred providers. This results in incentives for healthcare providers to improve the quality and reduce the price of care. One of the instruments to guide enrollees to preferred providers is by providing healthcare advice. In order to use healthcare advice as an effective instrument, it is important that enrollees accept the health insurer as a healthcare advisor. As trust in health insurers is not high, this may be an obstacle for enrollees to be receptive to the health insurer’s advice. This study aims to investigate the association between trust in the health insurer and the willingness to receive healthcare advice from the health insurer in the Netherlands. In terms of receiving healthcare advice, we examine both enrollees' willingness to approach the health insurer themselves and their willingness to be approached by the health insurer.

**Methods:**

In February 2021, a questionnaire was sent to a representative sample of the Dutch population. The questionnaire was completed by 885 respondents (response rate 59%). Respondents were asked about their willingness to receive healthcare advice, and trust in the health insurer was measured using a validated multiple item scale. Logistic regression models were conducted to analyse the results.

**Results:**

Enrollees with more trust in the health insurer were more willing to approach their health insurer for healthcare advice (OR = 1.07, *p* = 0.00). In addition, a higher level of trust in the health insurer is significantly associated with the odds that enrollees would like it/really appreciate it if their health insurer actively approached them with healthcare advice (OR = 1.07, *p* = 0.00). The role of trust in the willingness to receive healthcare advice is not proven to differ between groups with regard to educational levels, health status or age.

**Conclusions:**

This study confirms that trust plays a role in the willingness to receive healthcare advice from the health insurer. The association between the two emphasizes the importance to increase enrollees’ trust in the health insurer. As a result, health insurers may be better able to fulfil their role as healthcare advisor.

## Background

The healthcare systems of several countries, such as the Netherlands, Switzerland, Israel and Germany, are based on the theory of managed competition [1–3]. This theory aims to improve the quality of care and contain increasing healthcare costs by stimulating competition between third-party purchasers and care providers [1, 4–6]. In such a healthcare system third-party purchasers, mostly health insurers, purchase care on behalf of their enrollees [5]. Enrollees have the option to switch health insurers. As a result, health insurers have an interest in offering enrollees a more attractive health plan than competitors [1, 5–7]. Therefore insurers negotiate with healthcare providers on price and quality of care. In order for health insurers to have a strong position during these negotiations, it is crucial that they are able to guide their enrollees to preferred providers [7–10]. In that case healthcare providers must seriously consider the possibility that an insurer and their enrollees switch to a competitor [7, 10]. According to the theory of managed competition this gives care providers an incentive to improve the quality of care and reduce the price of care [4, 7, 10].

The ability to channel enrollees is thus indispensable for the functioning of a healthcare system with managed competition. This study focuses on the healthcare system of the Netherlands, of which the general principles of the health insurance system can be found in Table 1. The focus in this article is on basic health insurance. In the Netherlands citizens aged 18 years and older are obliged to take out basic health insurance. In 2022, citizens could choose from 60 health insurance policies offered by 20 health insurers divided into 10 health insurance groups [11]. All insurers in the Netherlands are private companies. They offer the same, extensive, basic health insurance package, of which the content is determined by the government. Nevertheless, health insurance policies can differ in terms of conditions and premium enrollees have to pay. For instance, because Dutch health insurers can apply selective contracting [5, 7]. This means that health insurers do not contract all healthcare providers. When enrollees choose a health insurance policy with restrictive conditions, which are usually offered at a lower premium, care from non-contracted providers is not always fully reimbursed. This makes it financially less attractive for these enrollees to visit healthcare providers that are not contracted by the health insurer. Health insurers can therefore use selective contracting as an instruments to guide enrollees to preferred providers.

**Table 1:** General principles of the Dutch Health Insurance Act

• Citizens aged 18 years and older are obliged to take out health insurance for basic health insurance [12]
• Everyone aged 18 years and older contributes to the cost of healthcare through premiums, contributions and taxes [13]
• Each year citizens are free to choose between the different health insurance policies offered by private health insurers [13]
• The content of the basic health insurance package is determined by the government, and includes many necessary medical care, medicines and aids [12]
• There is a deductible: the first €385 of healthcare costs must be paid by the enrollee, except for GP consultations, maternity care, home nursing and care for children under 18 years. In addition, enrollees can choose for a voluntary deductible up to €500 per person per year in exchange for a lower premium [14]
• An enrollee can take out supplementary insurance for care not included in the basic health insurance package. For example, reimbursement for treatment at the dentist or physiotherapist [12]
Roles of health insurers
• Health insurers are obliged to accept everyone for the basic health insurance, no matter health status or other characteristics [12]. Furthermore, the enrollee’s personal situation does not affect the health insurance premium. This means that the insured's health, age or income make no difference to the amount of the premium [12]. Through a risk equalization model, health insurers are financially compensated for predictable variation in individual medical expenses [15]
• Health insurers have a duty of care, meaning that their enrollees must have access to all care from the basic health insurance package within a reasonable time and travel distance. Health insurers must therefore purchase sufficient care or mediate if someone cannot get to a healthcare provider quickly enough (waiting time mediation) [12]
• Health insurers are expected to provide good information to their enrollees on policies, costs, reimbursements and waiting times for care and support [12]

Another way to guide enrollees to preferred providers is to provide healthcare advice [16]. Healthcare advice can consist of different services, such as waiting list mediation, advice on the most suitable care provider, advice and guidance in arranging care or obtaining medical aids as well as in arranging a second opinion, advice aimed at prevention and health promotion, and assistance in preparing a meeting with a doctor [17]. Although healthcare advice currently consists mainly of answering questions from the insured, a number of health insurers indicate that, if legislation allows, they would like to do more in terms of actively approaching insured with advice [18]. Actively approaching enrollees can be done in various ways, such as by phone calls or sending letters and e-mails [18]. By providing advice, especially with services on guidance to the most appropriate healthcare provider, insurers can guide their enrollees to visit the providers with whom they have made agreements on price, quality and/or volume of care. Although literature shows that, in practice, health insurers had limited focus on quality in their contractual negotiations with providers so far [19], increasing their advisory role may ensure that health insurers will be incentivized to pay more attention to quality of care in addition to volume and prices. This, in turn, could give providers incentives for both price reduction and quality improvement of care [4, 7, 10]. Besides that, healthcare advice could also lead to a better match between the needs of enrollees and the care offered.

In order to use healthcare advice as an effective instrument within the healthcare system to guide enrollees to preferred providers, it is important that enrollees embrace the health insurer as a healthcare advisor. However, literature shows that a large group of Dutch enrollees is not open to healthcare advice from the health insurer [16, 20]. A survey-based study showed that slightly more than half of the respondents indicated that they would not like it if their health insurer took on an advisory role in choosing a healthcare provider [20]. Another Dutch study showed that only about a third of the enrollees indicated that they would approach their health insurer for advice on a suitable healthcare provider [16]. Enrollees' trust in the health insurer may play a role in this [16, 20, 21]. Low trust in health insurers could be a bottleneck for enrollees to be open to advice from the health insurer. Literature shows that trust in health insurers in the Netherlands is low [22, 23]. Reasons for low trust might be, for example, the lack of health insurance literacy, the fact that health insurers are seen as for-profit organisations, the belief that third-parties should not interfere in the doctor-patient relationship, and media influencing the public opinion about health insurers [24]. Furthermore, there is a so-called ‘credible commitment’ problem, which means that health insurers have a problem convincing consumers that they are committed to contracting high-quality care rather than merely the least expensive care [21]. In addition, we know from a Dutch study that trust of enrollees specifically in the advisory role of the health insurer is not high [25]. As a result, low trust could threaten the functioning of the healthcare system based on the theory of managed competition, because the health insurer would be less able to use healthcare advice as an instrument to eventually influence the price and/or quality of care. Furthermore, when enrollees do not trust the healthcare advice from the health insurer and do not follow it, it is difficult for health insurers to align care to the needs of enrollees.

Although a number of studies have already been conducted into trust in health insurers [20, 22, 25], the association between trust in the health insurer and the willingness to receive healthcare advice has not been studied before. This study therefore focuses on the association between trust in the health insurer and the willingness to receive healthcare advice from the health insurer in the Netherlands. In terms of receiving healthcare advice, we examine both enrollees' willingness to approach the health insurer themselves and their willingness to be approached by the health insurer. Finding an association would emphasize the importance of taking actions to build trust in the health insurer so that the Dutch healthcare system, in which health insurers have been assigned the role of healthcare advisors, can function more optimal. We also investigate whether the role of trust in the willingness to receive healthcare advice is stronger for certain groups of people. In this study the concept of healthcare advice is defined by four types of advice: advice on what the most suitable care provider is, waiting list mediation, guidance in arranging care, and assistance in preparing a meeting with a doctor. We answer two research questions in this study: ‘What is the relationship between trust in the health insurer and the willingness of enrollees to receive healthcare advice from their health insurer?’. And: ‘Is the relationship between trust in the health insurer and the willingness of enrollees to receive healthcare advice from their health insurer different for certain groups of people?

### Hypotheses

Enrollees’ need for healthcare advice stems from a lack of knowledge or information. For example, enrollees do not have experience with all care providers to assess them for quality. The health insurer is expected to have the data, knowledge and expertise to inform enrollees by giving healthcare advice. However, because the insurer has more information than the enrollee, there is a certain information asymmetry [26, 27]. This information asymmetry may make it difficult for the enrollee to assess the quality of the health insurer's advice, in particular if the insurer is not fully transparent about the data or information on which they base their advice. This may create uncertainty which leads to a risk of receiving a less appropriate advice. The enrollee must believe that the insurer is honest in its advice and will have their best interest at heart. We know from literature that trust reduces perceived risk [28–30]. Therefore our expectation is that enrollees are more willing to receive advice from the health insurer when they have more trust in the health insurer, because this may reduce the perceived risk of receiving a less appropriate advice. Hence we expect that trust in the health insurer is an important precondition for their enrollees’ willingness to receive healthcare advice.

H1: Enrollees who have more trust in their health insurer will be more willing to receive healthcare advice from their health insurer.

The perceived risk of receiving a less appropriate advice may differ between groups. For instance, low-educated enrollees may have a greater perceived risk of receiving bad healthcare advice than high-educated enrollees, as low-educated enrollees are expected to have less knowledge and skills to assess the health insurer's advice [31]. As a result, they may have a greater information asymmetry compared to the high-educated enrollees, which leads to more uncertainty about the health insurer's advice. We therefore expect that the relationship between trust and the willingness to receive healthcare advice is different for groups of people with different educational levels. As we know that trust reduces perceived risk [28–30], we expect that the level of trust has a greater impact on the willingness to receive healthcare advice for people with a lower level of education than people with a higher level of education.

H2: The role of trust in the willingness to receive healthcare advice is stronger for lower educated people than for higher educated people.

Furthermore, health status may influence the role of trust in the willingness to receive healthcare advice. This arises from the perception that people with a poor health status are in a vulnerable position as they tend to have more complex care needs. As a result, people with a poor health condition are expected to have a greater perceived risk to receive a less appropriate advice by their health insurer about a suitable treatment or healthcare provider. We therefore expect that the relationship between trust and the willingness to receive healthcare advice is different for people with a different health status. Because trust reduces perceived risk [28–30], we expect that the level of trust has a greater impact on the willingness to receive healthcare advice for people with a poorer health status than for people with a better health status.

H3: The role of trust in the willingness to receive healthcare advice is stronger for people with poorer health than for those with better health.

Following the same reasoning as for health status, age may also influence the role of trust in the willingness to receive healthcare advice. Older people may have a greater perceived risk of receiving a less appropriate healthcare advice, as they are more likely to have (multiple) chronic conditions, which may make their care needs more complex. We expect that the level of trust has a greater impact on the willingness to receive healthcare advice for older than for younger people, as trust reduces perceived risk [28–30].

H4: The role of trust in the willingness to receive healthcare advice is stronger for older than for younger people.

## Methods

### Data

Data were collected using the Dutch Health Care Consumer Panel, an access panel managed by Nivel (the Netherlands Institute for Health Services Research) [32]. This panel collects opinions on, knowledge about and experiences with healthcare in the Netherlands among citizens. At the time of the study, February 2021, the panel had approximately 11,500 members from the general Dutch population aged 18 years and older. Their background characteristics such as age, sex and educational level are registered. The panel can only be joined through invitation, it is not possible for people to sign up on their own initiative. Members agree to be asked to participate in surveys on a regular basis. The data are analysed pseudonymized, and processed according to the panel’s privacy policy, which complies with the General Data Protection Regulation (GDPR). According to Dutch legislation, neither obtaining informed consent, nor approval by a medical ethics committee, is obligatory for carrying out research using the panel [32]. Participation is voluntary and members are not forced to participate in surveys, or to answer questions within the surveys. They can stop their membership at any time without giving a reason.

### Questionnaire

In February 2021, a questionnaire was sent to a sample of 1,500 panel members, representative of the adult population in the Netherlands with regard to age and sex. The questionnaire was developed by the authors, who have expertise on this research topic and in conducting survey-based research. Besides questions on other topics related to the choice of health insurance, the questionnaire included questions on enrollees' willingness to receive healthcare advice, a validated question on trust in the health insurer, and a question about their health status. The concept version of the questionnaire was submitted to the programme committee of the Nivel Dutch Health Care Consumer Panel, who had the opportunity to give feedback. This committee consists of representatives of different stakeholders in the healthcare sector, including the Dutch Consumers Association, and ‘Zorgverzekeraars Nederland’, the umbrella organisation of health insurers. There were no comments from the committee, so the content of the questionnaire did not change. The questionnaire could be filled in online or by post depending on the personal preference of the panel members. The panel members could fill in the questionnaire from the 9^th^ of February until the 11^th^ of March 2021. Completing the questionnaire took respondents approximately 15 to 20 min. Two reminders were sent to respondents who had not yet filled in the online questionnaire at that time and one reminder to respondents who had not yet filled in the paper questionnaire. The questionnaire was completed by 885 panel members (response rate 59%).

### Measures

#### The willingness of enrollees to receive healthcare advice

The willingness of enrollees to receive healthcare advice from their health insurer was measured by two questions. Before the questions were presented, respondents were introduced to the concept of healthcare advice through mentioning four examples: advice on what the most suitable care provider is, waiting list mediation, guidance in arranging care, and assistance in preparing a meeting with a doctor.

The first question was: ‘Suppose you need one of the above-mentioned types of advice. Would you approach your health insurer about this?’ The response categories on this question were: 1. ‘definitely not’, 2. ‘probably not’, 3. ‘probably yes’, 4. ‘definitely yes’, and 5. ‘I do not know’. To make the measure suitable for analysis, a dummy variable was created (1 = probably/definitely yes, 0 = probably/definitely not). The answer option 'I don't know' has been converted into a missing value (*n* = 93).

The second question was: ‘What would you think if your health insurer actively approached you with advice, for example about the quality of a specific care provider?’ The response categories on this question were: 1. ‘I would find that very unpleasant’, 2. ‘I would find that unpleasant’, 3. ‘I would like that’, 4.’ I would really appreciate that’, and 5. ‘I have no opinion about that’. Also this measure was transformed into a dummy variable, in order to make it suitable for the analysis (1 = I would like that/I would really appreciate that, 0 = I would find that (very) unpleasant). The answer option ‘I have no opinion about that’ has been converted into a missing value (*n* = 157).

#### Trust in the health insurer

Trust in health insurers was measured by the Health Insurer Trust Scale (HITS), a validated scale to measure patients' trust in health insurers, developed by Zheng et al. [33]. According to Zheng et al. [33], this scale is based on a conceptual model that assumes that insurer trust has four components that reflect overlapping aspects of insurance organizations: 1. fidelity—caring for the subject's interests or welfare, 2. competence—making correct decisions and avoiding mistakes, 3. honesty—telling the truth and avoiding intentional falsehoods, and 4. confidentiality—proper use of sensitive information. The scale consists of 11 items for which the response categories are strongly agree (score 5), agree (score 4), neutral (score 3), disagree (score 2), and strongly disagree (score 1) (see Table 2). The scoring is reversed in the case of a negative question (items 2, 4–7, and 9). Trust is measured by the sum of the 11 item scores ranging from 11 to 55. A higher score indicates more trust. A score has only been calculated if respondents have responded to all the statements of the scale. The scores of respondents who completed the scale partially (*n* = 24) or not at all (*n* = 56) were converted to missing scores. For this study we used the validated Dutch translation of this scale by Hendriks et al. [34].

**Table 2:** Health Insurer Trust Scale (HITS) (Zheng et al., 2002)

	**Items**	**Response categories**
1	You think the people at your health insurer are completely honest	strongly agree (score 5); agree (score 4); neutral (score 3); disagree (score 2); strongly disagree (score 1)
2	Your health insurer cares more about saving money than about getting you the treatment you need	strongly agree (score 1); agree (score 2); neutral (score 3); disagree (score 4); strongly disagree (score 5)
3	As far as you know, the people at your health insurer are very good at what they do	strongly agree (score 5); agree (score 4); neutral (score 3); disagree (score 2); strongly disagree (score 1)
4	If someone at your health insurer made a serious mistake, you think they would try to hide it	strongly agree (score 1); agree (score 2); neutral (score 3); disagree (score 4); strongly disagree (score 5)
5	You feel like you have to double check everything your health insurer does	strongly agree (score 1); agree (score 2); neutral (score 3); disagree (score 4); strongly disagree (score 5)
6	You worry that private information your health insurer has about you could be used against you	strongly agree (score 1); agree (score 2); neutral (score 3); disagree (score 4); strongly disagree (score 5)
7	You worry there are a lot of loopholes in what your health insurer covers that you don't know about	strongly agree (score 1); agree (score 2); neutral (score 3); disagree (score 4); strongly disagree (score 5)
8	You believe your health insurer will pay for everything it is supposed to, even really expensive treatments	strongly agree (score 5); agree (score 4); neutral (score 3); disagree (score 2); strongly disagree (score 1)
9	If you got really sick, you are afraid your health insurer might try to stop covering you altogether	strongly agree (score 1); agree (score 2); neutral (score 3); disagree (score 4); strongly disagree (score 5)
10	If you have a question, you think your health insurer will give a straight answer	strongly agree (score 5); agree (score 4); neutral (score 3); disagree (score 2); strongly disagree (score 1)
11	All in all, you have complete trust in your health insurer	strongly agree (score 5); agree (score 4); neutral (score 3); disagree (score 2); strongly disagree (score 1)

#### Background variables

The background variables that are known from the panel members and are included concern: age (continuous), gender (0 = male, 1 = female), educational level (1 = low (none, primary school or pre-vocational education), 2 = middle (secondary or vocational education), 3 = high (professional higher education or university)), and self-reported health status (1 = bad/fair, 2 = good, 3 = very good/excellent).

### Data analysis

Descriptive statistics were computed to describe the characteristics of the study population. Logistic regression models were applied in order to test the hypotheses. The two measures for the willingness of enrollees to receive healthcare advice from their health insurer were analysed in separate models. We examined the main and the interaction effects. Through the interaction effects we investigated the hypotheses (H2, H3 and H4) that state that the relationship between trust and the willingness to receive healthcare advice is modified by educational level, age and health status. A significance level of 5% (*p* ≤ 0.05) was maintained for these analyses. All analyses were performed using STATA version 16.1.

## Results

### Descriptives

Table 3 presents the descriptive statistics. The male/female ratio in the total group of 885 respondents was 48/52. Respondents were on average 54 years old. 47% of the respondents were highly educated. 17% rated their health as bad or fair. On average, respondents had a score of 37 (range 13–54) on the Health Insurer Trust Scale. Fifty nine percent of the respondents would probably or definitely approach their health insurer about the mentioned types of advice, and 46% would like it/really appreciate it if their health insurer actively approached them with healthcare advice.

**Table 3:** Descriptive statistics of the respondents

	**Number of respondents (n)**	**Percentage (%) or mean (SD)**
**Gender**	**885**	
Male	429	48%
Female	456	52%
**Age**	**885**	54 (16.53)
18–39 years	238	27%
40–64 years	418	47%
65 years and older	229	26%
**Education**	**873**	
Low (none, primary school or pre-vocational education)	93	11%
Middle (secondary or vocational education)	367	42%
High (professional higher education or university)	413	47%
**Health (self-reported)**	**829**	
Bad/fair	144	17%
Good	384	46%
Very good/excellent	301	36%
**Health Insurer Trust Scale** (range 11–55)	**805**	37 (5.78) (range: 13–54)
**Suppose you need one of the above-mentioned types of advice**^**1**^**. Would you approach your health insurer about this?**	**745**	
Definitely/probably not	304	41%
Definitely/probably yes	441	59%
**What would you think if your health insurer actively approached you with advice**^**1**^**, for example about the quality of a specific care provider?**	**685**	
(Very) unpleasant	368	54%
I would like that/ really appreciate that	317	46%

^1^ advice on what the most suitable care provider, waiting list mediation, guidance in arranging care or assistance in preparing a meeting with a doctor

### Testing hypothesis 1

Model 1 (see Table 4) examines the relationship between enrollees’ trust in the health insurer and their willingness to approach their health insurer for healthcare advice. Enrollees with more trust in the health insurer were more willing to approach their health insurer for healthcare advice (OR = 1.07). This result is in line with H1. Besides that, Model 1 shows that older enrollees and enrollees with a middle level of education are more willing to approach their health insurer for healthcare advice than younger enrollees and enrollees with a low or high level of education.

**Table 4:** Multivariate logistic regression to examine the associations between Q1 and trust

Q1: Suppose you need one of the above-mentioned types of advice. Would you approach your health insurer about this? (1 = definitely/probably yes, 0 = definitely/probably not)									
		**Model 1 (*n* = *699)***		**Model 2 (*n* = *699)***		**Model 3 (*n* = *699)***		**Model 4 (*n* = *699)***	
		**Odds Ratio**	***P*-value**	**Odds Ratio**	***P*-value**	**Odds Ratio**	***P*-value**	**Odds Ratio**	***P*-value**
Trust (HITS)^**^		1.07	0.00^*^	1.02	0.77	1.16	0.00^*^	1.07	0.00^*^
**Gender**	Male	Reference		Reference		Reference		Reference	
	Female	0.81	0.20	0.82	0.23	0.80	0.18	0.81	0.20
Age^**^		1.01	0.04^*^	1.01	0.04^*^	1.01	0.03^*^	1.01	0.03^*^
Education^***^	Low	Reference		Reference		Reference		Reference	
	Middle	2.00	0.02^*^	2.01	0.02^*^	2.02	0.02^*^	2.02	0.02^*^
	High	1.28	0.40	1.29	0.38	1.31	0.36	1.31	0.36
**Health (self-reported)**	Bad/fair	Reference		Reference		Reference		Reference	
	Good	0.94	0.79	0.94	0.80	0.82	0.44	0.95	0.82
	Very good/excellent	0.74	0.23	0.75	0.24	0.66	0.11	0.74	0.23
Trust^*^education	Low			Reference					
	Middle			1.05	0.44				
	High			1.07	0.26				
Trust^*^health	Bad/fair					Reference			
	Good					0.90	0.02^*^		
	Very good/excellent					0.92	0.05		
Trust^*^age								1.00	0.31
**Constant**		1.25	0.48	1.23	0.52	1.41	0.29	1.23	0.51

^*^ Significant *p*-value

^**^ Centered around the mean

^***^ Low = none, primary school or pre-vocational education. Middle = secondary or vocational education. High = professional higher education or university

Furthermore, Model 5 (see Table 5) examines the relationship between trust in the health insurer and the willingness to be actively approached by their health insurer with healthcare advice. In line with H1, a higher level of trust in the health insurer is significantly associated with the odds that enrollees would like it/really appreciate it if their health insurer actively approached them with healthcare advice (OR = 1.07). In combination with the result of model 1, H1 is therefore accepted. Beyond that, Model 5 shows that men and those in bad/fair health are more likely to like it/really appreciate it if their health insurer actively approached them with healthcare advice than women and those in very good/excellent health.

**Table 5:** Multivariate logistic regression to examine the associations between Q2 and trust

Q2: What would you think if your health insurer actively approached you with advice, for example about the quality of a specific care provider? (1 = I would like that/really appreciate that, 0 = I would find that (very) unpleasant)									
		**Model 5 (*n* = *642)***		**Model 6 (*n* = *642)***		**Model 7 (*n* = *642)***		**Model 8 (*n* = *642)***	
		**Odds Ratio**	***P*-value**	**Odds Ratio**	***P*-value**	**Odds Ratio**	***P*-value**	**Odds Ratio**	***P*-value**
Trust (HITS)^**^		1.07	0.00^*^	0.97	0.62	1.05	0.09	1.07	0.00^*^
**Gender**	Male	Reference		Reference		Reference		Reference	
	Female	0.41	0.00^*^	0.41	0.00^*^	0.42	0.00^*^	0.41	0.00^*^
Age^**^		1.00	0.43	1.00	0.43	1.00	0.39	1.01	0.30
Education^***^	Low	Reference		Reference		Reference		Reference	
	Middle	1.29	0.41	1.26	0.46	1.31	0.39	1.32	0.38
	High	0.60	0.11	0.58	0.09	0.61	0.11	0.62	0.13
**Health (self-reported)**	Bad/fair	Reference		Reference		Reference		Reference	
	Good	0.83	0.42	0.83	0.45	0.85	0.49	0.84	0.47
	Very good/excellent	0.57	0.03^*^	0.58	0.04^*^	0.57	0.03^*^	0.57	0.03^*^
Trust^*^education	Low			Reference					
	Middle			1.12	0.07				
	High			1.10	0.11				
Trust^*^health	Bad/fair					Reference			
	Good					1.01	0.79		
	Very good/excellent					1.04	0.30		
Trust^*^age								1.00	0.09
**Constant**		1.97	0.04^*^	2.01	0.04^*^	1.90	0.05	1.93	0.05^*^

^*^ Significant *p*-value

^**^ Centered around the mean

^***^ Low = none, primary school or pre-vocational education. Middle = secondary or vocational education. High = professional higher education or university

### Testing hypothesis 2

Model 2 (see Table 4) examines whether the role of trust in their willingness to approach their health insurer for healthcare advice is stronger for lower educated people than for higher educated people. The role of trust in the willingness to receive healthcare advice does not significantly differ between people with a low level of education and those with a middle or high level of education. Furthermore, Model 6 (see Table 5) examines whether the role of trust in the willingness to be actively approached by their health insurer with healthcare advice is stronger for lower educated people than for higher educated people. The role of trust in the willingness to be actively approached by their health insurer with healthcare advice does not differ between people with a low, a middle or high level of education. H2 is therefore rejected.

### Testing hypothesis 3

Model 3 (see Table 4) examines whether the role of trust in the willingness to approach their health insurer for healthcare advice is stronger for people with a poorer health status than for those with a better health status. In line with H3, the role of trust in the willingness to receive healthcare advice is stronger for people in bad/fair health than for those in good health (OR = 0.90). For the association between trust and the willingness to receive healthcare advice between people in bad/fair health and people in very good/excellent health we find a nearly significant effect (*p* = 0.052). Additionally, Model 7 (see Table 5) examines whether the role of trust in the willingness to be actively approached by their health insurer with healthcare advice is stronger for people with a poorer health status than for those with a better health status. The role of trust in the willingness to be actively approached by their health insurer with healthcare advice does not differ between people in bad/fair health and those in good or very good/excellent health. H3 is therefore neither accepted nor rejected, given the different findings from the two models.

### Testing hypothesis 4

Model 4 (see Table 4) examines whether the role of trust in the willingness to approach their health insurer for healthcare advice is stronger for older than for younger people. The role of trust in the willingness to receive healthcare advice is not significantly different for older than for younger people. In addition, Model 8 (see Table 5) examines whether the role of trust in the willingness to be actively approached by their health insurer with healthcare advice is stronger for older than for younger people. The role of trust does not differ between younger and older people. Therefore, H4 is rejected.

## Discussion

In accordance with our first hypothesis, our study shows there is an association between trust in the health insurer and the willingness to receive healthcare advice from the health insurer. We found that when enrollees have more trust in the health insurer, they are more willing to approach them for healthcare advice, and are also more positive about being actively approached by their health insurer. This finding makes it plausible that trust can remove the perceived risk of receiving less appropriate advice. In the presence of a higher level of trust, the uncertainty about the advice, caused by the information asymmetry between the health insurer and the enrollee seeking care, seems to be reduced. Our study shows an average score of 37 on the Health Insurer Trust Scale (HITS), which we consider as moderate trust. This score is about the same as that measured in an older study from 2007 that used the HITS to measure trust in health insurers in general in the Netherlands and found an average score of 36.6 [34]. Since we also know from other literature that the current level of trust in health insurers in the Netherlands is not high [23, 24], the results of our study emphasize the importance of investigating ways to increase trust in health insurers. By increasing enrollees’ trust in their health insurer, the proportion of enrollees who are receptive to healthcare advice may increase. As a result health insurers will have the opportunity to take on their role of health care advisor. This might lead to a better functioning of the healthcare system based on the theory of managed competition, as healthcare advice can align care with the needs of enrollees and as it creates a greater ability to guide enrollees to preferred providers.

The current distrust towards health insurers could be explained by different theories. The first theory is based on the principle that many citizens have little knowledge of the role of insurers in the healthcare system, also referred to as the ‘lack-of information model’ by Maarse et Jeurissen [24]. The distrust resulting from this theory may be eliminated by better disclosure of the role of insurers, however the literature on this is still limited [24, 25]. Further research could focus on enrollees’ knowledge of insurers' roles and the relationship of this to trust. The second theory is based on the principle that there is a critical attitude to competition in healthcare among citizens, arising from the idea that health insurers are profit-driven organisations who do not act in the interests of the patient [24]. Maarse et. al. [24] call this theory ‘the anti-competition model’. More transparency about the quality of the selected providers may increase trust in health insurers [22]. Additional research could provide insight as to whether transparency is a good instrument for increasing trust in health insurers. Thirdly, distrust may come from the idea that insurers should not interfere in the relationship between doctor and patient [24]. According to Maarse et al. [24], who refer to this theory as the ‘pro-profession model’, this idea may change by building a trust relationship between insurers and the providers of medical care. They assume that doctors’ trust in health insurance will positively affect the patients’ understanding of the insurers’ role in health care [24]. Finally, distrust may come from the bad publicity insurers usually get in the media [24, 35]. Maarse et al. [24] call this the ‘political communication model’. It is difficult for the health insurer to influence the information brought by third parties, such as the media [36]. However, showing good behavior as an insurer can improve the personal experiences of both insured and health care providers and thus avoid negative media coverage [36]. In addition, trust in the health insurer may be increased by not ignoring negative reporting and criticism, but by responding to it openly and honestly. It could also help to bring out positive reports about the achievements of the health insurer in advancing the interests of the enrollees.

Our results show that there is an association between trust in the health insurer and the willingness to receive healthcare advice from the health insurer. The degree of this association is not proven to be different between groups. It has not been found that for certain groups, with regard to educational levels, health status or age, an increase in trust in the health insurer is in particular important. The finding that no difference in association was found based on educational level could possibly be explained by the fact that a lack of publicly available quality information that is useful for the enrollee [19, 37]. This makes it also difficult for those with a high level of education to examine and assess the healthcare advice they receive. As a result, all enrollees might have about the same information asymmetry. The result that no difference in association has been proven based on health status is still arguable, as one of the models showed a significant result, while the other model did not. In future research a comparable analysis could be performed again to clarify this.

Apart from the association with trust, the results of our study show that men were more open than woman to being approached by the health insurer with advice. Since literature shows that men generally show a delay in help-seeking when they become ill [38–40], our result that men are relatively more open to healthcare advice from the health insurer might imply a solution to promote earlier healthcare utilization among men through this advice. Furthermore, our results show that younger people were less likely to approach their health insurer for healthcare advice than older people. In addition, respondents in very good or excellent health were in particular less open to being approached with advice from their health insurer. An explanation might be that healthcare plays a less important role in life for most young people and for those in good health. Therefore this result is not surprising and not a concern, as it is especially important that those who use care regularly are open to healthcare advice.

### Strenghts and limitations

A strength of this study is that it contributes to the limited research on the field of healthcare advice from the health insurer. A strength of the method of the study is that trust in health insurers was measured by a validated scale (HITS). The use of this scale contributes to the validity of this study. Furthermore, the questionnaires were sent both by post and online. As a result, people who are less digitally literate could also participate in the survey.

A limitation of this study is that the respondents were not entirely representative of the Dutch population of enrollees. The respondents are relatively older and higher educated compared to the general Dutch population [41, 42], as younger and less educated people were underrepresented. However, we expect that this did not affect our regression results, since all subgroups are of sufficient size to perform association analyses.

Another limitation of this study is that there might be other factors besides trust and the enrollees’ characteristics which are not included in this study, but may influence enrollees’ willingness to receive healthcare advice. An important factor might be earlier experience with healthcare advice. Enrollees who have previously received healthcare advice and had a negative experience with it, will be less open to it than enrollees who have had a positive experience. Unfortunately, since the assessment of previous experiences of healthcare advice was not measured in the questionnaire, we were not able to include this factor in the analyses. Future studies could focus on other factors that may influence enrollees’ willingness to receive healthcare advice.

A third limitation is that a part of the enrollees may be not familiar with what healthcare advice from the health insurer entails. In addition, healthcare advice is a broad concept. It could be that enrollees are positive about certain forms of healthcare advice, but for other forms they are not. This may affect the results, as it may also make it more difficult for respondents to assess whether they would be open to it. However, we introduced the topic in the questionnaire by mentioning examples of healthcare advice, and therefore assume that the participants were able to make a good assessment of whether or not they would be willing to receive healthcare advice. The same applies to the concept of being ‘actively approached’. This can be carried out in several manners, such as by phone calls or sending letters and e-mails. The distinction between enrollees’ openness to the different manners cannot be made from this research. For this study we looked at healthcare advice in a broad sense, because we were interested in enrollees’ attitude towards the advisory role of the health insurer in general. Further research might focus separately on specific forms of healthcare advice in a variety of manners.

## Conclusions

This study confirms that trust plays a role in the willingness to receive healthcare advice from the health insurer. The association between the two emphasizes the importance of high trust in the health insurer. The role of trust is not proven to be different between specific groups. We find a general relationship between trust and openness to healthcare advice, independent of the presumed degree of information asymmetry or the interest people may have in receiving appropriate advice. This study can be seen as a first exploration of this association, to contribute to the knowledge about the importance of trust in the health insurer for the functioning of healthcare systems based on the theory of managed competition. The results are relevant for countries in which the health insurers have also been attributed a role as a healthcare advisor. Further research could focus on possibilities to increase trust in health insurers, as this may increase enrollees’ receptiveness to healthcare advice from the health insurer. As a result, health insurers may be better able to fulfil their assigned role as healthcare advisor.

## Data Availability

The datasets generated and/or analysed during the current study are not publicly available, as it was collected by means of the Dutch Health Care Consumer Panel. The Dutch Health Care Consumer Panel has a program committee, which supervises processing the data and decides about the use of the data. This program committee consists of representatives of the Dutch Ministry of Health, Welfare and Sport, and other key stakeholders in the organisation of healthcare in the Netherlands. All research conducted within the Consumer Panel has to be approved by this program committee. The committee assesses whether a specific research fits within the aim of the Consumer Panel, that is strengthen the position of the health care user. Data derived from the Consumer Panel is only available upon request from Prof. Judith de Jong, PhD, project leader of the Dutch Health Care Consumer Panel.

## References

[1] Enthoven AC, van de Ven WP (2007). Going Dutch—managed-competition health insurance in the Netherlands. N Engl J Med.

[2] Van de Ven WP, Beck K, Buchner F, Schokkaert E, Schut FE, Shmueli A (2013). Preconditions for efficiency and affordability in competitive healthcare markets: are they fulfilled in Belgium, Germany, Israel, the Netherlands and Switzerland?. Health Policy.

[3] Glazer J, McGuire TG (2011). Gold and silver health plans: accommodating demand heterogeneity in managed competition. J Health Econ.

[4] Enthoven AC (1993). The history and principles of managed competition. Health Aff (Millwood).

[5] Bes RE. Selective contracting by health insurers: the perspective of enrolees: Maastricht University; 2018.

[6] Victoor A, Brabers A, van Esch T, de Jong J (2019). An assessment of the Dutch experience with health insurers acting as healthcare advisors. PLoS ONE.

[7] Bes RE, Curfs EC, Groenewegen PP, de Jong JD (2017). Selective contracting and channelling patients to preferred providers: a scoping review. Health Policy.

[8] Wu VY (2009). Managed care's price bargaining with hospitals. J Health Econ.

[9] Sorensen AT (2003). Insurer-hospital bargaining: negotiated discounts in post-deregulation connecticut. J Ind Econ.

[10] Varkevisser M, Polman N, Van der Geest S (2006). Zorgverzekeraars moeten patiënten kunnen sturen [Health insurers should be able to guide patients]. Economisch Statische Berichten.

[11] Kerncijfers zorgverzekeraars [Key figures health insurers]: Nederlandse Zorgautoriteit; 2022 [Available from: https://www.nza.nl/zorgsectoren/zorgverzekeraars/kerncijfers-zorgverzekeraars.

[12] Zvw-algemeen: Hoe werkt de Zorgverzekeringswet [Health Insurance Act in general: how the Health Insurance Act works]: Zorginstituut Nederland; [Available from: https://www.zorginstituutnederland.nl/Verzekerde+zorg/zvw-algemeen-hoe-werkt-de-zorgverzekeringswet.

[13] Het Zorgverzekeringsfonds (Zvf) [The Health Insurance Fund]: Zorginstituut Nederland; [Available from: https://www.zorginstituutnederland.nl/financiering/fondsbeheer-zvf-en-flz-en-subsidies/zorgverzekeringsfonds.

[14] Eigen risico (Zvw) [Deductible (Health Insurance Act)]: Zorginstituut Nederland; 2022 [Available from: https://www.zorginstituutnederland.nl/Verzekerde+zorg/eigen-risico-zvw#:~:text=Vrijwillig%20eigen%20risico,-De%20zorgverzekeraar%20kan&text=Het%20vrijwillige%20eigen%20risico%20kan,bovenop%20het%20verplicht%20eigen%20risico.

[15] Van Kleef RC, Van Vliet RC, Van de Ven WP (2013). Risk equalization in the Netherlands: an empirical evaluation. Expert Rev Pharmacoecon Outcomes Res.

[16] Victoor A, Potappel A, de Jong J. Zorgadvies door zorgverzekeraars [Healthcare advice by health insurers]. 2019.

[17] Van Esch T, Brabers A, M K, De Jong J. Monitor overstapseizoen 2017–2018: een onderzoek naar informatievoorziening door de zorgverzekeraar, potentiële overstapbelemmeringen voor verzekerden en de zorgverzekeraar als zorgadviseur. [Monitor switching season 2017–2018: a survey on health insurer information provision, potential barriers to switching for enrollees and the health insurer as healthcare advisor]. Utrecht: Nivel; 2018.

[18] Holst L, van der Hulst F, Brabers A, de Jong J. Kennisvraag: De zorgverzekeraar als adviseur [Expertise question: The health insurer as advisor]. 2022.

[19] Stolper KC, Boonen LH, Schut FT, Varkevisser M (2019). Managed competition in the Netherlands: do insurers have incentives to steer on quality?. Health Policy.

[20] Bes R, Wendel S, de Jong JD (2012). Het vertrouwensprobleem van zorgverzekeraars [The trust problem of health insurers]. ESB.

[21] Boonen LH, Schut FT (2011). Preferred providers and the credible commitment problem in health insurance: first experiences with the implementation of managed competition in the Dutch health care system. Health Econ Policy Law.

[22] Boonen L, Schut E (2009). Zorgverzekeraars kampen met vertrouwensprobleem [Health insurers face trust problem]. Economisch-Statistische Berichten.

[23] Meijer MA, Brabers, A.E.M. & Jong, J.D. de. Barometer Vertrouwen in de Gezondheidszorg [Barometer of Trust in Healthcare] Utrecht: Nivel; 2022 [Available from: https://www.nivel.nl/nl/consumentenpanel-gezondheidszorg/resultaten-vertrouwen.

[24] Maarse H, Jeurissen P (2019). Low institutional trust in health insurers in Dutch health care. Health Policy.

[25] Hoefman RJ, Brabers A, De Jong J. Vertrouwen in zorgverzekeraars hangt samen met opvatting over rol zorgverzekeraars. 2015.

[26] Retchin SM (2007). Overcoming information asymmetry in consumer-directed health plans. Am J Manag Care.

[27] D’Cruz MJ, Kini RB. The effect of information asymmetry on consumer driven health plans. Integration and Innovation Orient to E-Society Volume 1: Springer; 2007. p. 353–62.

[28] Pavlou PA (2003). Consumer acceptance of electronic commerce: Integrating trust and risk with the technology acceptance model. Int J Electron Commer.

[29] Pavlou PA, Gefen D (2004). Building effective online marketplaces with institution-based trust. Inf Syst Res.

[30] Das TK, Teng B-S (2001). Trust, control, and risk in strategic alliances: an integrated framework. Organ Stud.

[31] Jacobs W, Amuta AO, Jeon KC (2017). Health information seeking in the digital age: an analysis of health information seeking behavior among US adults. Cogent Social Sciences.

[32] Brabers A, de Jong J. Nivel Consumentenpanel Gezondheidszorg: basisrapport met informatie over het panel 2022 [Nivel Health Care Consumer Panel: core report with information on the panel 2022]. 2022.

[33] Zheng B, Hall MA, Dugan E, Kidd KE, Levine D (2002). Development of a scale to measure patients' trust in health insurers. Health Serv Res.

[34] Hendriks M, Delnoij DM, Groenewegen PP. Het meten van vertrouwen in de zorgverzekeraar: psychometrische eigenschappen van een Nederlandse vragenlijst [Measuring trust in the health insurer: psychometric properties of a Dutch questionnaire]. TSG. 2007;85(5):280–6.

[35] Van der Schee E. Public trust in health care: Exploring the mechanisms: NIVEL; 2016.

[36] Groenewegen PP, Hansen J, De Jong JD (2019). Trust in times of health reform. Health Policy.

[37] Springvloet L, Rolink M, Bos N, Zagt A, de Jong J, Friele R, et al. De Transparantiemonitor [The Transparency Monitor]. 2021.

[38] Galdas PM, Cheater F, Marshall P (2005). Men and health help-seeking behaviour: literature review. J Adv Nurs.

[39] Smith J, Braunack-Mayer A, Wittert G. What do we know about men's help-seeking and health service use? 2006. 10.5694/j.1326-5377.2006.tb00124.x 16411874

[40] Yousaf O, Grunfeld EA, Hunter MS (2015). A systematic review of the factors associated with delays in medical and psychological help-seeking among men. Health Psychol Rev.

[41] Bevolking; geslacht, leeftijd en nationaliteit op 1 januari [Population; gender, age and nationality on 1 January]. In: CBS, editor. 2021.

[42] Bevolking; hoogstbehaald onderwijsniveau en onderwijsrichting [Population; highest level of education attained and direction of education]. In: CBS, editor. 2022.

